# Attenuated relationship between salivary oxytocin levels and attention to social information in adolescents and adults with autism spectrum disorder: a comparative study

**DOI:** 10.1186/s12991-020-00287-2

**Published:** 2020-06-05

**Authors:** T. Fujioka, T. X. Fujisawa, K. Inohara, Y. Okamoto, Y. Matsumura, K. J. Tsuchiya, T. Katayama, T. Munesue, A. Tomoda, Y. Wada, H. Kosaka

**Affiliations:** 1 Faculty of Education, University of Fukui, Fukui, Fukui Japan; 2 Department of Child Development, United Graduate School of Child Development, Osaka University, Kanazawa University, Hamamatsu University School of Medicine, Chiba University, and University of Fukui, Suita, Osaka Japan; 3 Research Center for Child Mental Development, University of Fukui, Eiheiji, Fukui Japan; 4 College of Liberal Arts and Sciences, Kitasato University, Sagamihara, Kanagawa, Japan; 5 Department of Neuropsychiatry, Faculty of Medical Sciences, University of Fukui, Eiheiji, Fukui Japan; 6 Waseda Institute for Advanced Study, Waseda University, Shinjuku, Tokyo Japan; 7 Research Center for Child Mental Development, Hamamatsu University School of Medicine, Hamamatsu, Shizuoka Japan; 8 Department of Psychiatry, Hamamatsu University School of Medicine, Hamamatsu, Shizuoka Japan; 9 Kaga Mental Hospital, Kaga, Ishikawa Japan

**Keywords:** Autism spectrum disorder, Oxytocin, Social information, Eye-tracking, Adolescents, Adult

## Abstract

**Background:**

Previous research studies have assessed the relationship between attention to social information and peripheral (e.g., plasma and salivary) oxytocin (OT) levels in typically developing (TD) children and children with autism spectrum disorder (ASD). A relationship between them was observed in TD children, but not in children with ASD. However, this relationship remains unexamined in other age groups. To clarify whether this lack of association is maintained throughout development in individuals with ASD, we aimed to assess the relationship between salivary OT levels and attention to social information in adolescents and adults with and without ASD.

**Methods:**

We recruited male adolescents and adults with ASD (*n* = 17) and TD participants (*n* = 24). Using the all-in-one eye-tracking system Gazefinder, we measured the percentage fixation time allocated to social information. We also measured the salivary OT levels and Autism Spectrum Quotient (AQ) of participants. Subsequently, we confirmed group differences and conducted a correlation analysis to investigate the relationships between these three measures.

**Results:**

Salivary OT levels did not show any significant difference between the ASD and TD groups and were negatively correlated with the AQ in the whole-group analysis, but not in within-group analysis. Individuals with ASD had significantly lower percentage fixation times than did TD individuals for eye regions in human faces with/without mouth motion, for upright biological motion, and for people regions in the people and geometry movies. The percentage of fixation for geometric shapes in the people and geometry movies was significantly higher in the ASD than in the TD group. In the TD group, salivary OT levels were positively correlated with percentage fixation times for upright biological motion and people and negatively correlated with inverted biological motion and geometry. However, no significant correlations were found in the ASD group.

**Conclusions:**

Our exploratory results suggest that salivary OT levels in adolescents and adults with ASD are less indicative of attention to social stimuli than they are in TD adolescents and adults. It is suggested that their association is slightly weaker in adolescents and adults with ASD and that this attenuated relationship appears to be maintained throughout development.

## Background

Autism spectrum disorder (ASD) is a neurodevelopmental disorder characterized by a “deficit of social communication and social interaction” and “restricted, repetitive patterns of behavior, interests, or activities”, which comprise various specific symptoms [1]. Although the clinical presentation of ASD is modified by appropriate support and reasonable accommodation for certain difficulties, features persist throughout life. Previous studies have assessed these features across development to better understand the clinical presentation of ASD.

Abnormal attention to social information, a component of sociality, is observed in ASD throughout various developmental stages. As first reported by Kanner [2], gaze fixation deficits are one of the most representative features of ASD and many researchers have since confirmed this objectively using eye-tracking methodology. For example, individuals with ASD tend to gaze less at the face [3–10], especially at the eye region [3, 10–19], look less at biological motion [20–22], and pay greater attention to geometric shapes when people and geometric shapes are presented simultaneously [11, 23–26] than do typically developed (TD) individuals. These unique gaze patterns for the face, eyes, and geometry have been reported in both children [3–5, 7–10, 13–15, 19, 23–26] and adults [6, 11, 12, 16–19], although the unique gaze pattern for biological motion was reported only in children. Furthermore, fixation times for social information have also been shown to be negatively correlated with the severity of social deficits [12, 14, 15, 25]. Therefore, abnormal attention to social information is observed over a wide age range and reflects the severity of social deficits.

Oxytocin (OT), a neuropeptide released from the posterior pituitary gland, is likely to be associated with sociality, including abnormalities in attention to social information. OT underlies maternal behavior, sexual behavior, anxiolysis, social preference and recognition [27], and symptoms of mental disorders [28]. Central OT is measured in the cerebrospinal fluid collected by a lumbar puncture [29, 30]. However, this method is highly invasive. Therefore, many researchers have measured peripheral OT levels in the blood (plasma and serum) or saliva as a proxy for central OT levels [31–33]. In fact, significant correlations have been observed between the central OT and plasma OT levels [30] and between plasma OT and salivary OT levels [31, 34–36]. Note, however, that a meta-analysis reported that the relationship between the central OT and plasma OT levels was limited [37], and one study failed to show any significant correlation between plasma OT and salivary OT levels [38]. Furthermore, studies have reported inconsistent results regarding the relationship between central and peripheral OT levels [30, 39], and the validity of this proxy remains under debate [40]. Despite these limitations, plasma and salivary OT levels have been found to be positively correlated with sociality scores (the higher the score, the higher the sociality) using various tests in both children [41] and adults [31, 42]. Furthermore, in TD adults, serum OT levels were found to be negatively correlated with the imagination subscale score of the Autism Spectrum Quotient (AQ) [43]. These findings suggest that OT plays a role in the systems that regulate sociality and that peripheral OT levels also reflect the degree of social deficits.

An abnormal relationship between peripheral OT levels and sociality has been reported in individuals with ASD. One study reported that the more severe the social deficits, the lower the plasma OT levels in children with ASD [44]. However, in children with ASD, other studies have shown no correlations or an inverse relationship between plasma OT levels and the severity of social deficits [41, 45–48]. In adults with ASD, the plasma OT levels were not correlated with ASD traits [49]. In sum, these results suggest that the association between OT and the degree of social deficits seen in TD individuals appears to be attenuated in individuals with ASD.

The relationship between attention to social information, an aspect of sociality, and peripheral OT levels has only been investigated in children. Such studies have reported that the salivary OT levels are positively correlated with gaze fixation on the eye area in TD infants and children [50] and on object areas in a finger-pointing movie in preschool TD children [51]. However, only one study investigated individuals with ASD, reporting that salivary OT levels did not correlate with attention to social information in preschool children with ASD [51]. These results suggest that the association between peripheral OT levels and gaze patterns for social information may be attenuated in children with ASD.

However, no previous study has assessed the relationship between peripheral OT levels and gaze patterns in other age groups. Therefore, as the relationship between attention to social information and peripheral OT levels was not investigated other than in preschool children in a previous study [51], it remains unclear whether the attenuated association that is observed in children with ASD persists into adolescence and adulthood or whether it changes throughout development. As attention to social information is a lifelong prominent feature of ASD, understanding the relationship between peripheral OT and gaze for social information at various developmental stages could shed light on deficits characterizing ASD in individuals of different ages. Therefore, to clarify whether adolescents and adults with ASD also show an attenuated association between attention to social information and peripheral OT levels, as observed in children with ASD, we aimed to investigate the extent to which salivary OT levels affect the patterns of gazing at social information in adolescents and adults with and without ASD.

## Methods

### Participants

We recruited 17 adolescent and adult men with ASD and 24 TD male participants. In this study, we only included men because reported variations in OT levels in women are higher [52].

Participants with ASD (aged 16 to 39 years old) were recruited from the Department of Neuropsychiatry, University of Fukui Hospital, Japan, and the Department of Psychiatry and Neurobiology, Kanazawa University Hospital, Japan. Two authors (TM and HK) diagnosed the participants based on the criteria of the Diagnostic and Statistical Manual of Mental Disorders, 5th Edition (DSM-5) [1] and the standardized criteria of the Diagnostic Interview for Social and Communication Disorders (DISCO) [53]. As OT appears to be associated with symptoms of various mental disorders [28], we recruited individuals who were only diagnosed with ASD; thus, participants were not included if they met the diagnostic criteria for any other psychopathology, e.g., attention-deficit/hyperactivity disorder, schizophrenia spectrum disorders, depressive disorders, anxiety disorders, or obsessive–compulsive disorder. Fifteen individuals with ASD completed the Wechsler Adult Intelligence Scale-Third Edition (WAIS-III) [54] and all scored ≥ 70 on the full-scale IQ (FSIQ). Two individuals with ASD refused to complete the WAIS-III, but were considered to have an average IQ based on their past or current academic performance.

TD participants (aged 20 to 53 years) were recruited from the local community. Individuals with a history of major medical or neurological illnesses, including epilepsy, significant head trauma, or a lifetime history of alcohol or drug dependence were excluded from the study. Although we did not measure the IQ in the TD group, all TD participants had graduated from high school or college.

### Salivary OT measurement

Saliva samples were collected using Salivettes^®^ (Sarstedt, Rommelsdorft, Germany). Saliva samples were frozen and stored at − 80 °C in the laboratory. Before assaying, saliva samples were lyophilized overnight and incubated at − 20 °C to concentrate them four times. The dry samples were reconstructed in the assay buffer immediately before analysis using an OT enzyme immunoassay commercial kit without extraction (Assay Designs Inc., Ann Arbor, MI). Although extraction is considered necessary to measure peripheral OT levels using an enzyme immunoassay [55], measurement without extraction was validated using a solid methodology [56, 57]. The kit’s sensitivity was 15 pg/ml. Each sample was duplicated and levels were calculated using the SpectraMax^®^ micro-plate reader (Molecular Device, Sunnyvale, CA), according to relevant standard curves. The average intra- and inter-assay coefficients of variation (CV) were 4.6% and 6.1%, respectively.

### Attention to social information measurement

We used Gazefinder to assess attention to social information. Gazefinder is an all-in-one eye-tracking system used to evaluate the percentage fixation times allocated to specific objects on a video monitor [11, 50, 51]. Participants’ eye positions were measured using infrared light sources and cameras located below a 19-in. thin-film transistor (1280 pixels × 1024 pixels). Using corneal reflection techniques, eye position was recorded as (X, Y) coordinates at a frequency of 50 Hz (i.e., 3000 data collections/min). Calibration of eye position recordings was performed using a five-point method.

**Fig. 1:**
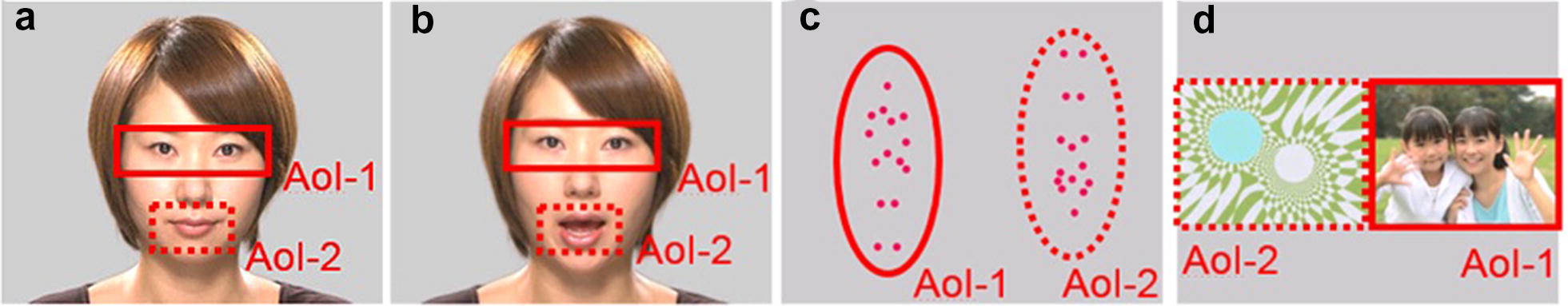
Gazefinder^®^ movie samples and their areas-of-interest (AoIs). **a** Screenshot of human face without mouth motion, AoI-1 and AoI-2 include the eye and mouth regions, respectively; **b** screenshot of human face with mouth motion, AoI-1 and AoI-2 include the eye and mouth regions, respectively; **c** screenshot of biological motion, AoI-1 and AoI-2 are the upright and inverted images, respectively; **d** screenshot of people and geometry, AoI-1 and AoI-2 are people and geometry, respectively

Following calibration, the Gazefinder presented four types of movies: (A) human faces without mouth motion; (B) human faces with mouth motion; (C) biological motion of a human, and (D) people and geometry. (A) Human faces without mouth motion included movies of a still face (7 s), of eyes blinking (an actress repeatedly opens and closes her eyes for 7 s), and of a silent face (an actress with a still face appears for 3 s, and this movie is presented after the *mouth moving* face movie described below). (B) Human faces with mouth motion included movies of a *mouth moving* face (an actress repeatedly opens and closes her mouth for 4 s) and of a *talking* face (7 s). In the *talking* face movie, the actress says, “Konnichiwa” (“Hello”), “Onamaewa?” (“What is your name?”), and “Issyoniasobouyo” (“Let’s play together”). (C) Biological motion presented upright and inverted biological motion simultaneously for 20 s. The movie was accompanied by the song “Under the Big Chestnut Tree” to which an upright human danced. (D) The people and geometry movies consisted of movie of people and geometric shapes presented at the same time and at the same size (16 s) and a movie of geometric shapes depicted in small-frame images in a small window on a movie of people (16 s). The former contains four pairs of people and geometric shape movies, each lasting 4 s, and the latter contains two types of movies lasting 8 s. The presentation order of these stimuli was randomly predetermined; all participants saw the stimuli in the same order. Figure 1 presents samples of the stimuli.

Percentage fixation times allocated to areas-of interest (AoIs) on the video monitor, which are the areas considered to contain social information and set by default, were automatically calculated (time allocated to a particular area/duration of stimulus presentation), and the results could be shown immediately after the videos were viewed. Figure 1 shows the AoIs for each stimulus. Human face movies included two AoIs (i.e., AoI-1 was the eye region and AoI-2 was the mouth region). Biological motion movies included two AoIs (i.e., AoI-1 was upright biological motion involving dancing to the song, and AoI-2 was inverted biological motion that was presented upside down and played backward relative to the upright biological motion). For the movie of people and geometry, there were two AoIs (i.e., AoI-1 was the area with people, and AoI-2 was the area with geometry). In addition to these AoIs, the percentage fixation time allocated to areas other than AoI(s) (i.e., area outside the AoI) was also calculated. Figure 1 presents the AoIs of the stimuli.

### Procedures

Testing was carried out in a silent room in the Department of Neuropsychiatry, University of Fukui Hospital. First, saliva samples were collected only once. Participants then watched the movies while their gaze was detected using Gazefinder. Finally, all participants completed the AQ to determine the severity of ASD [58]. The AQ comprises 50 questions, with scores ranging from 0 to 50. Higher AQ scores indicate higher severity of ASD (Table 1). The study protocol was approved by the ethics committee of the University of Fukui and conformed to the tenets of the Declaration of Helsinki (as revised in 2000). After a complete explanation of the study, all participants or their parents/legal guardians provided written informed consent.

**Table 1:** Participant characteristics

	ASD (*n* = 17)	TD (*n* = 24)	*t*	*p*
Age (years)	27.4 ± 7.2	29.0 ± 9.8	.54	.590
WAIS-III				
Full-scale IQ	100.8 ± 16.3	−		
Verbal IQ	104.9 ± 17.7	−		
Performance IQ	95.6 ± 16.6	−		
AQ				
Total	30.9 ± 6.4	16.3 ± 6.4	7.00	< .001

### Data analysis

According to previous research [51], we excluded participants for whom the available percentage fixation time was < 50% (i.e., Gazefinder could not detect eye position more than 50% of the time). One participant with ASD, for whom the available percentage fixation time was 31%, was excluded. Therefore, data were available for statistical analysis for 16 individuals with ASD and 24 TD individuals.

At first, we carried out the Shapiro–Wilk test of normality for the salivary OT level and found that it was normally distributed (*W* = .952, *p* = .092). Therefore, we used parametric tests in the subsequent analysis. To clarify group differences in salivary OT levels and the percentage fixation times for each AoI in Gazefinder, we investigated differences between the ASD and TD groups using two-tailed *t* test. For the AoIs, as in the previous study using Gazefinder with adults with ASD [11], to avoid type 1 statistical errors, we applied Bonferroni corrections and set .05 divided by the number of AoIs as the significance level for each item; we set .025 (.05/2) for all stimuli. We subsequently conducted a correlation analysis to clarify the relationships among salivary OT levels, AQ, and the percentage fixation times to the AoI for each movie. For AoIs, to avoid type 1 statistical error, we applied Bonferroni correction and set .025 (.05/2) as the significance level for the correlation analysis as described above.

Subsequently, we investigated whether there were significant differences in correlation coefficients between the ASD and TD groups. All statistical analyses were conducted using IBM SPSS statistical software, version 24 (IBM Corp., Armonk, NY).

## Results

### Demographic data

There was no significant difference in the mean ages between the ASD and TD groups (*t* (39) = .54, *p* = .590). The AQ scores were significantly different between the ASD and TD groups (*t* (39) = 7.00, *p* < .001). These scores for both groups and the IQ scores for the ASD group are summarized in Table 1.

### Between-groups analysis

**Fig. 2:**
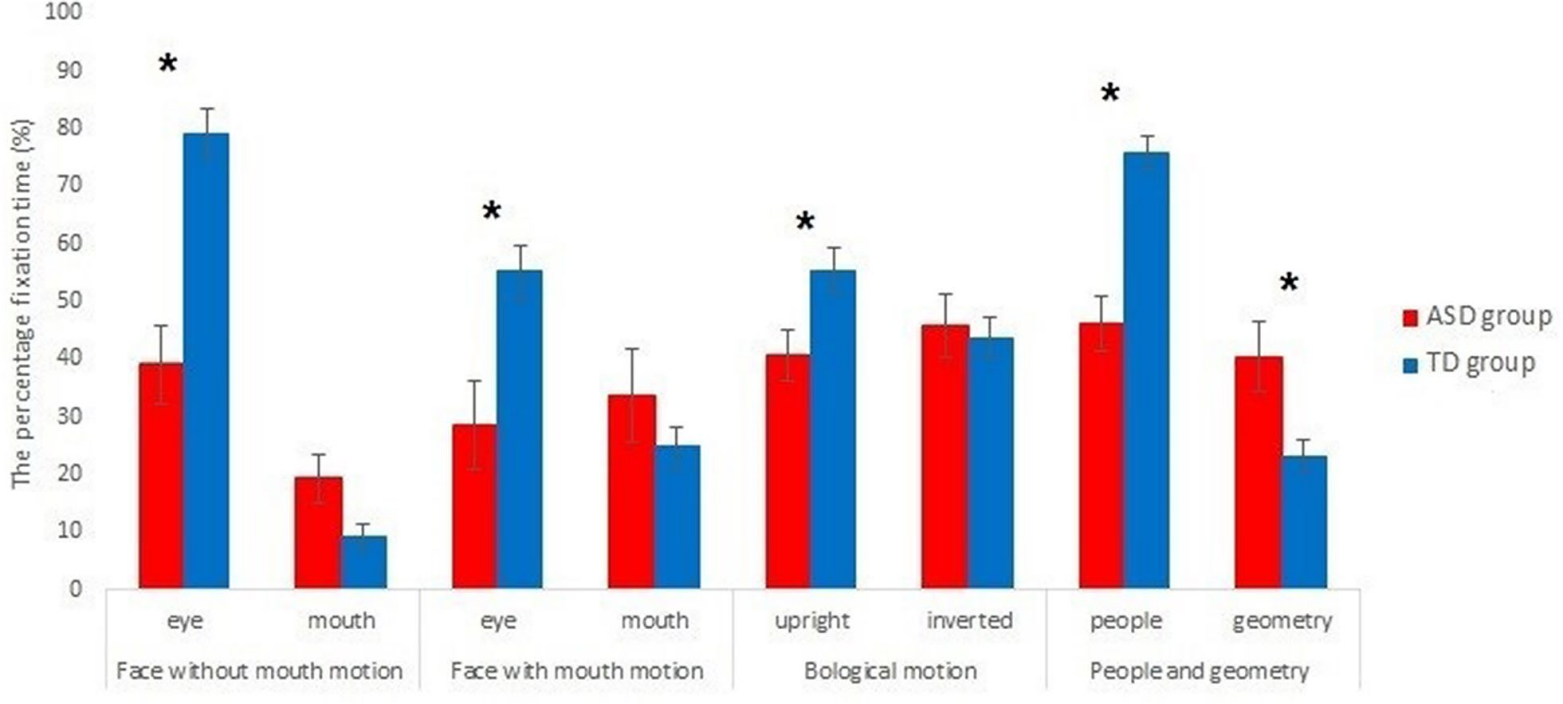
Bar graphs of the percentage fixation times and standard errors of each area-of-interest (AoI). The red and blue bars indicate the percentage fixation times of the ASD group and TD group, respectively. Error bars indicate standard errors of the mean. *p < .025 (= .05/2)

There was no significant difference in salivary OT levels between the groups (ASD 36.2 ± 13.2 pg/ml, TD 43.6 ± 17.0 pg/ml, *t* (38) = 1.45, *p* = .154, Cohen’s d = .49). Individuals with ASD had significantly lower percentage fixation times than did TD individuals for eye regions in human faces with/without mouth motion and for people regions in the people and geometry movies. On the one hand, the percentage of fixation for geometric shapes in the people and geometry movies was significantly higher in the ASD than in the TD group (Table 2 and Fig. 2).

**Table 2:** Group differences in mean fixation percentages

	ASD		TD		Group difference		Effect size	
	Mean	SD	Mean	SD	*t*	*p*	Cohen’s *d*	
Face								
Without mouth motion								
% Eye	39.1	27.5	79.1	20.6	5.26	< .001*	1.74	b
% Mouth	19.4	16.5	9.1	10.8	2.19	.039	.79	a
With mouth motion								
% Eye	28.6	30.5	55.1	22.0	3.20	.003*	1.06	b
% Mouth	33.8	32.4	24.8	17.6	1.01	.323	.38	
Biological motion								
% Upright	40.7	17.7	55.4	18.7	2.48	.018*	.82	b
% Inverted	45.7	21.7	43.6	18.6	.32	.747	.11	
People and geometry								
% People	46.3	19.1	75.7	13.8	5.67	< .001*	1.88	b
% Geometry	40.4	24.5	23.2	13.8	2.56	.018*	.94	b

* *p* < .025 (= .05/2)

^a^Moderate effect size (> .50)

^b^High effect size (> .80) [83]

### Correlation analysis of salivary OT levels

**Fig. 3:**
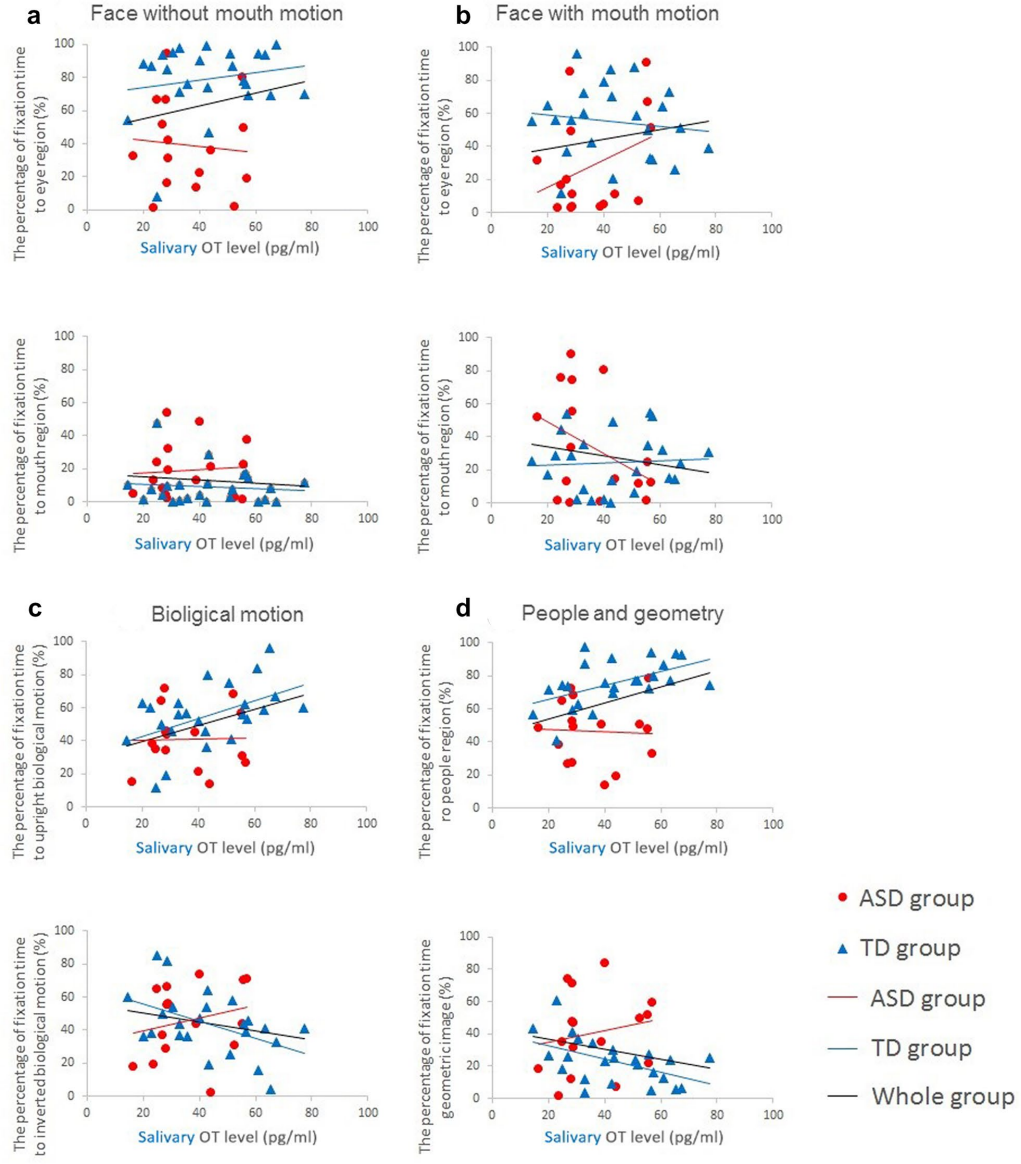
Scatter plots of the correlation between the percentage of fixation time to area-of-interest (AoI) and salivary oxytocin (OT) level. Red circles and lines are data from the ASD group, and blue triangles and lines are from the TD group. The black line is the approximate curve for all groups

First, salivary OT levels and AQ for the whole group were found to be significantly correlated (*r* = − .398, *p* = .011). However, correlation analysis revealed no significant correlation between salivary OT level and AQ for both the ASD and TD groups (*r* = − .361, *p* = .169 and *r* = − .349, *p* = .095, respectively). The results of the correlation analysis between salivary OT levels and the AoIs in Gazefinder are shown in Table 3, and Fig. 3 shows the scatter plots of these results. Salivary OT levels were significantly positively correlated with percentage fixation times for upright biological motion and the people region in the people and geometry movies. These significant correlations were observed in an analysis of the whole group, as well as in a subgroup analysis of the TD group. Furthermore, there were significant negative correlations between salivary OT levels and the percentage fixation times for inverted biological motion and the geometric regions in the people and geometry movies in the TD group.

**Table 3:** Correlations between salivary oxytocin (OT) level and the percentage fixation times

	Correlation with OT level					
	Whole group		ASD		TD	
	*r*	*p*	*r*	*p*	*r*	*p*
Face						
Without mouth motion						
% Eye	.205	.204	− .083	.760	.187	.381
% Mouth	− .106	.516	.081	.765	− .110	.608
With mouth motion						
% Eye	.163	.314	.355	.178	− .130	.544
% Mouth	− .177	.274	− .390	.136	.062	.772
Biological motion						
% Upright	*.394*	.012*	.021	.939	*.496*	.014*
% Inverted	− .212	.189	.239	.374	− *.473*	.020*
People and geometry						
% People	*.357*	.024*	− .048	.859	*.531*	.008*
% Geometry	− .241	.133	.193	.473	− *.514*	.010*

* *p* < .025 (= .05/2)

### Correlation analysis for AQ

For the whole-group correlation analysis, AQ was significantly negatively correlated with percentage fixation times for the eye region for faces without/with mouth motion and the people region in the people and geometry movies and positively correlated with the mouth region for faces without/with mouth motion and the geometry region in the people and geometry movies. For the ASD group, correlation analysis showed that the mouth region for faces with mouth motion and inverted biological motion were significantly positively correlated with AQ. For the TD group, AQ was negatively correlated with the people region and positively correlated with the geometry region in people and geometry movies. The results of the correlation analysis between AQ and the AoIs in Gazefinder are shown in Table 4.

**Table 4:** Correlations between the Autism Spectrum Quotient (AQ) and the percentage fixation times

	Correlation with AQ					
	Whole group		ASD		TD	
	*r*	*p*	*r*	*p*	*r*	*p*
Face						
Without mouth motion						
% Eye	− *.443*	.004*	.022	.935	.143	.504
% Mouth	*.358*	.023*	.442	.087	− .155	.469
With mouth motion						
% Eye	− *.390*	.013*	− .231	.390	.064	.766
% Mouth	*.396*	.011*	*.624*	.010*	.163	.446
Biological motion						
% Upright	− .250	.120	.066	.807	.036	.866
% Inverted	.223	.166	*.700*	.003*	− .049	.821
People and geometry						
% People	− *.604*	<.001*	.170	.529	− *.544*	.006*
% Geometry	*.617*	<.001*	.476	.062	*.581*	.003*

* *p* < .025 (= .05/2)

## Discussion

The aim of this study was to investigate the relationship between salivary OT levels and attention to social information in adolescents and adults with ASD and TD individuals. The salivary OT levels revealed no significant differences between the ASD and TD groups and were negatively correlated with the severity of ASD traits in the whole-group analysis, but not in the within-group analysis. The correlation analysis revealed that salivary OT level were positively correlated with percentage fixation times for upright biological motion and people regions in the people and geometry movies in whole-group and subgroup analyses of TD individuals. Moreover, OT levels were negatively correlated with percentage fixation times for inverted biological motion and geometric shapes in the people and geometry movies in TD individuals. In the ASD group, no significant correlations were observed between peripheral OT levels and percentage fixation times for any item.

### Salivary OT levels in the ASD group

There was no significant difference in salivary OT levels between individuals with ASD and TD participants. Although some studies have reported lower peripheral OT levels in ASD [41, 44, 45, 59, 60], others have reported similar or higher peripheral OT levels in individuals with ASD than in TD individuals [46–48, 51, 61]. In particular, studies of adults with ASD have reported mixed results, with both lower [60] and higher [61] peripheral OT levels. A meta-analysis published in 2016 found no evidence of peripheral OT levels being impaired in ASD [62]. Summarizing the above, researchers have not obtained consistent results for peripheral OT levels in ASD, which appear to be affected by many factors other than diagnosis. This complexity may have influenced our results.

Several factors can affect OT levels in ASD. One factor may be the ASD subgroup, as suggested by Lee et al. [63], who indicated that “the decrease of the oxytocin receptor gene expression is related to some subgroups of ASD”. This statement was based on the results of Gregory et al. [64], which showed that in some samples of individuals with ASD, there was significantly greater methylation of the oxytocin receptor gene and reduced oxytocin mRNA at postmortem analysis. The report by Modahl et al. [41], in which plasma OT levels have been shown to correlate with some ASD traits in an “aloof” subgroup but not in an “active-but-odd” subgroup, may support the existence of subgroups of ASD. In summary, taken together, these findings indicate that some, but not all, individuals with ASD show abnormal peripheral OT levels. Therefore, the existence of ASD subgroups within our group of ASD study participants may have affected the results of the current study in which the ASD group showed moderately, but not significantly lower OT levels than the TD group. In addition to the potential ASD subgroups as a factor, some studies have found that OT levels are positively correlated with anxiety [65, 66] and negatively correlated with depression [67]. Although the underlying biological mechanisms remain unknown, OT appears to be released for the purpose of reducing the amygdala and hypothalamic–pituitary–adrenocortical reactivity to stressors [68]. Anxiety and depression disorders are highly comorbid with ASD [69, 70], and these factors may disrupt OT regulation in ASD. As previously mentioned, the traits of the ASD subgroups or those of other mental disorders may have affected the results of previous studies reporting on peripheral OT concentration levels.

What will be needed in future research based on this study’s results? Our participants in the ASD group were only diagnosed with ASD, and not with any other mental disorder, meaning that the participants in the ASD group did not show severe symptoms or traits of other mental disorders. Since we excluded participants with intellectual disability, our findings can be used to reflect characteristics exclusive to ASD, laying the foundation for future research. However, no genetic analysis or measures for ASD subgroups or psychological indices other than ASD were assessed in this study. Therefore, future studies are certainly needed to elucidate the influence of these factors.

### Relationship between salivary OT levels and percentage fixation times for social information

In TD individuals, salivary OT levels were correlated with percentage fixation times for upright and inverted biological motion and people and geometric regions in the people and geometry movies. OT is thought to modulate prosocial behavior with anxiolytic and anti-stress effects on the brain, especially in the amygdala [68, 71, 72]. The mechanisms underlying the prosocial effects of OT have been investigated [68], and, while it appears that OT may not have major effects on prosociality because it is modulated by many factors [73], higher peripheral OT levels do seem to lead to more social behavior [41–43, 51, 74]. This finding is consistent with our results showing that TD participants with high peripheral OT levels tend to gaze at more social information.

Interestingly, however, although salivary OT levels were significantly associated with percentage fixation times on the eyes and mouth in TD infants and children in a previous study [50], these results were not replicated in our study with TD adolescents and adults. This may have been because the TD adolescents and adults in our study might have frequently gazed at the eye region, therefore gazing at the mouth region less frequently, regardless of the salivary OT levels. As shown in Fig. 3, this was especially notable in movies that showed human face without mouth motion, which thus did not strongly lead the attention to the mouth [11, 50]; for these movies, even TD adolescents and adults who had low salivary OT levels gazed into the eye region at high rates. In fact, the percentage fixation times to the eye region in this study’s participants were higher than those of male children from a previous study, in which these averages were 66.89 ± 20.26% for faces without a moving mouth, and 25.98 ± 17.58% for faces with a moving mouth [50]. Taken together, these findings may indicate that all TD adolescent and adult participants in our study tended to gaze at the eye region in human faces more frequently than did TD children in previous studies. The eye region, which is related to intuiting the mental states of others [75, 76] and joint attention [77, 78], provides salient social information. Adolescents and adults may need to perform complex interactions in everyday life; thus, TD individuals may need to regularly pay more attention to the eye region. Therefore, TD adolescent and adult participants may gaze at the eye region even if their salivary OT levels are low, and significant associations between salivary OT levels and percentage fixation times on AoIs on human faces might not be observed. However, our results are unable to confirm our hypothesis, and further studies are warranted to further investigate the factors that affect gaze when seeking out social information, particularly in the context of eye region-centric gazes.

For individuals with ASD, we observed no significant correlations between salivary OT levels and gazing social information. This result pointed to the weak relationships between salivary OT levels and attention given to social information in ASD, which may be due to the same reason underlying the group differences in peripheral OT levels between groups (i.e., ASD subgroup, anxiety, depression, etc.). As described above, although we excluded individuals with comorbidities in the ASD group, we did not completely rule out these factors in this study; hence, future studies are warranted to fully clarify their influence. Previous studies on children with and without ASD have also reported significant correlations between salivary OT levels and fixation duration in TD children, but not in children with ASD [51]. Our results may thereby point to the continued disturbance of the association between salivary OT levels and fixation duration from preschool ages into adolescence and adulthood.

Significant correlations in the whole-group analysis were found in upright biological motion and the people region in people and geometry movies. Based on the concept underlying the term ‘spectrum’, which suggests that every individual has ASD tendencies to a certain degree, the salivary OT levels are presumed to be correlated with sociality, which spontaneously directs one’s eyes to social information. However, there were no significant correlations in face stimuli. As aforementioned, the eyes may be salient social stimuli and have special significance for everyday interpersonal relationships; therefore, TD individuals may also have gazed at the eye region, regardless of their salivary OT level.

In summary, the salivary OT levels appeared to be related to attention to social information in TD adolescents and adults, and this relationship appeared to be weaker in adolescents and adults with ASD. In the TD group, however, the types of social information that showed significant correlations with the salivary OT levels were different from those reported in the previous studies with children [50] described above. Hence, it is possible that the factors or mechanisms underlying the relationship in TD individuals, and its disturbance in ASD, between salivary OT levels and social attention may vary over the course of development. Therefore, longitudinal research is warranted in order to fully clarify the factors affecting them (the genetic or psychiatric factors described above) at various ages.

### Relationship between AQ and percentage fixation times for social information

Significant correlations in the whole-group analysis were found for all stimuli except for biological motion. These significant correlations may reflect group differences for AQ and percentage fixation times for social information. Regarding biological motion, there were no significant correlations despite the group difference. Biological motion was the only stimulus that did not include actual human beings in Gazefinder, and the quality of biological motion for social information stimuli may differ from that of others. It is a future task to clarify the factors related to the gaze of biological motion.

In the ASD group, AQ was significantly positively correlated with the percentage fixation times to the mouth region of faces with mouth motion and inverted biological motion. Previous studies have also reported that children and adults with ASD tend to pay attention to moving objects, not to salient social information [7, 11]. Consistent with previous findings, our results may reflect the fact that the stronger the characteristics of ASD, the more difficult it is to suppress attention to movement and redirect attention to social information.

In the TD group, AQ was significantly correlated with the percentage fixation times to people and geometry regions. In both cases, the stronger one’s ASD characteristics, the less the individual attended to social information. It was suggested that the simultaneous presentation of people and geometric patterns is a stimulus that can reflect the severity of ASD in TD adolescents and adults. Regarding the face stimuli, as described in “the relationship between salivary OT levels and percentage fixation times for social information”, TD adolescent and adult participants may gaze at the eye region, regardless of their salivary OT levels, and the association between ASD severity and social attention to the eyes may be disrupted. In biological motion, as described above, as the quality of the biological motion for social information stimuli may differ from that of others’, and some factors other than ASD traits more strongly affect attention than biological motion, the correlation between them may not be significant.

### Relationship between salivary OT levels and AQ

A significant correlation between salivary OT level and AQ was observed in the whole-group analysis. The significant correlation in the whole-group analysis was presumed to reflect the fact that the ASD subgroup showed abnormal peripheral OT levels, that is, the result reflecting the difference between the ASD and TD groups. Based on the spectrum concept described above, the salivary OT level reflects the ASD characteristics in the whole-group analysis. Conversely, the significant correlations between salivary OT level and AQ were not observed in the within-group analysis. In a previous study, in TD adults, the salivary OT level was not correlated with the AQ total score, while it was negatively correlated with the AQ subscale “difficulties in imagination” [43]. Furthermore, in adults with ASD, the plasma OT levels were not correlated with ASD traits [49]. Our results of the within-group analysis are consistent with those reported by previous studies. Considering both the whole and within-group analyses in this study, it is appears that OT may not have major effect on autism traits or have major effect in only some individuals. Our results support the hypothesis that “it appears that OT may not have major effects on prosociality because it is modulated by many factors [73]”.

## Conclusion

We observed significant correlations between salivary OT levels and percentage fixation times for social information in TD adolescents and adults, but not in a group of adolescents and adults with ASD. This study’s results failed to demonstrate any significant differences in salivary OT levels between adolescents and adults with ASD without other mental disorders and TD individuals; however, the regulatory mechanisms underlying salivary OT levels and fixation durations may be attenuated in adolescents and adults with ASD. The ASD subgroups showed abnormal peripheral OT levels and/or some factors that did not strongly affect the TD group, which may have affected our results. In addition, the relationship between salivary OT levels and attention to social information was also weaker in preschool children with ASD than in TD children [51]; this weakened relationship may continue beyond childhood. However, the factors or mechanisms underlying this relationship remain unclear and may change throughout development. Furthermore, the lack of correlation between the eye region in face stimuli and salivary OT levels both in the ASD and TD groups is another interesting result, showing that the eye area may have special significance for both ASD and TD individuals. In future research, we will need to investigate what affects this attenuation in the ASD group and whether the causes of this relationship between peripheral OT levels and social attention in ASD and TD at different developmental stages are the same. By elucidating the nature of these factors, we will be able to better understand ASD symptoms and their development.

### Limitations and future directions

This study has some limitations that should be considered when interpreting its results. First, the sample size was relatively small. Future studies with larger sample sizes are required to confirm these results. Second, we did not exclude confounding factors that could have affected peripheral OT levels and/or gaze to social information. We speculate that these confounders may include a certain degree of heterogeneity within the ASD subgroups associated with genetic factors, anxiety, and depression. Studies adjusting for these confounding factors are warranted in order to confirm this study’s results. With regard to the TD group, we did not measure IQ. As such, in addition to controlling for the factors described above, it may be necessary to repeat the analysis in this study while controlling for IQ. Finally, some researchers have suggested that extraction is needed to measure peripheral OT levels using an enzyme immunoassay [55]. Although the method used in this study, without extraction, is a valid method for measuring peripheral OT levels [56, 57] and is widely used [31, 51, 79–82], future confirmation using extraction processing is warranted.

## Data Availability

The datasets used and/or analyzed during the current study are available from the corresponding author on reasonable request.

## Abbreviations

ADOS: Autism Diagnostic Observation Schedule
AoI: Area-of-interest
AQ: Autism Spectrum Quotient
ASD: Autism spectrum disorder
CARS: Childhood Autism Rating Scale
DISCO: Diagnostic Interview for Social and Communication Disorders
DSM: The Diagnostic and Statistical Manual of Mental Disorders
FSIQ: Full-Scale Intelligent Quotient
IQ: Intelligent Quotient
OT: Oxytocin
TD: Typically developed
VABS: Vineland Adaptive Behavior Scale
WAIS-III: Wechsler Adult Intelligence Scale—Third Edition
